# Does Subacromial Osteolysis Affect Shoulder Function after Clavicle Hook Plating?

**DOI:** 10.1155/2016/4085305

**Published:** 2016-02-29

**Authors:** Siwei Sun, Minfeng Gan, Han Sun, Guizhong Wu, Huilin Yang, Feng Zhou

**Affiliations:** Department of Orthopedic Surgery, The First Affiliated Hospital of Soochow University, No. 188 Shizi Street, Suzhou, Jiangsu 215006, China

## Abstract

*Purpose.* To evaluate whether subacromial osteolysis, one of the major complications of the clavicle hook plate procedure, affects shoulder function.* Methods.* We had performed a retrospective study of 72 patients diagnosed with a Neer II lateral clavicle fracture or Degree-III acromioclavicular joint dislocation in our hospital from July 2012 to December 2013. All these patients had undergone surgery with clavicle hook plate and were divided into two groups based on the occurrence of subacromial osteolysis. By using the Constant-Murley at the first follow-up visit after plates removal, we evaluated patients' shoulder function to judge if it has been affected by subacromial osteolysis.* Results.* We have analyzed clinical data for these 72 patients, which shows that there is no significant difference between group A (39 patients) and group B (33 patients) in age, gender, injury types or side, and shoulder function (the Constant-Murley scores are 93.38 ± 3.56 versus 94.24 ± 3.60, *P* > 0.05).* Conclusion.* The occurrence of subacromial osteolysis is not rare, and also it does not significantly affect shoulder function.

## 1. Introduction

In those cases of shoulder joint injuries, lateral clavicle fracture and acromioclavicular (AC) joint dislocation are the most common [1, 2]. Conventionally, these patients accepted conservative treatment mainly, as the clinical outcome was acceptable to most patients [3], excluding the unstable injury types such as Neer II fracture and Degree-III AC joint dislocation. With the development of the surgical technique, patients with the above injury types were increasingly advised by surgeon to accept surgery. Compared with conservative treatment, appropriate surgery can reduce the possibility of nonunion or deformed union happening [4–6]. The main surgery methods include K-wire, tension-band wiring, T-plate, and clavicle hook plate procedures.

The clavicle hook plate has been accepted gradually to treat lateral clavicle fractures (mainly for Neer II) and AC joint dislocations (Degree-III) [4, 5, 7–10]. Several studies have shown good outcome regarding bony union and shoulder function [4, 5, 7–13]. However, some complications could not be ignored in these studies, such as subacromial osteolysis, impingement symptoms, plate or screw fracture, and surrounding fracture. Among these main complications, subacromial osteolysis is a special complication in clavicle hook plate procedures and has a relatively higher incidence [4–6].

Studies about subacromial osteolysis are scarce. A portion of patients treated with the clavicle hook plate procedure in our hospital were reviewed to determine the occurrence of subacromial osteolysis in order to demonstrate whether the condition affects postoperation shoulder function.

## 2. Materials and Methods

### 2.1. Data Collection

In total, 72 patients were diagnosed as Neer II lateral clavicle fracture or Degree-III AC joint dislocation from July 2012 to December 2013 in our hospital. To exclude other potential effects, patients with old fracture or dislocation were not selected for this study (2 samples), combination of clavicle and scapula fracture was excepted (1 sample), and the patients who were lost follow-up were also eliminated (3 samples). All patients underwent a one-stage operation using a clavicle hook plate procedure within 1–3 days. Then the patients were asked to revisit the hospital for outpatient follow-up and take plain radiographs in the 1st, 3th, 6th, and 12th month after surgery. Finally, these 72 patients were divided into two groups: group A (with subacromial osteolysis) and group B (without subacromial osteolysis) according to the last X-ray results. Meanwhile their shoulder function was evaluated by Constant-Murley at the first revisit after the plates removed. Conventionally, in our hospital, the clavicle hook plate was removed approximately one year after it was implanted. Certainly, all the selected patients got clinical union in this study.

### 2.2. Surgical Technique

All operations were performed by the same surgical group. Firstly, the patients were supine on the operating table and received general anesthesia. The injured shoulder was typically raised before the beginning of surgery. Afterwards, a straight incision was made just medially to the AC joint over the fracture. The soft tissue which was inserted in the fracture site was removed and then exposed and reduced; large comminuted fragments were temporarily fixed with K-wires in some cases. We did not open the AC joint in any operation and incised part of the soft tissue under the acromion to insert the clavicle hook plate, without repairing the ligaments. The appropriate plate was selected and the hook was passed under the acromion posterior to the AC joint. The plate should be as close to the clavicle as possible, ensuring that the hook is under the acromion at a depth suitable for the C-arm. We used screws of the appropriate length and type according to the fracture pattern. Next, we fixed the screws and plate and checked the mobility of the shoulder. The incision was closed after hemostasis and irrigation, and a surgical drain could be placed and removed 1-2 days following surgery.

### 2.3. Statistical Analysis

Statistical analysis was performed with SPSS 19.0 statistical software (SPSS, Inc., Chicago, IL). Student's *t*-test and the chi-square analysis method were used to evaluate the difference between the two groups. *P* < 0.05 was considered statistically significant.

## 3. Results

**Figure 1:**
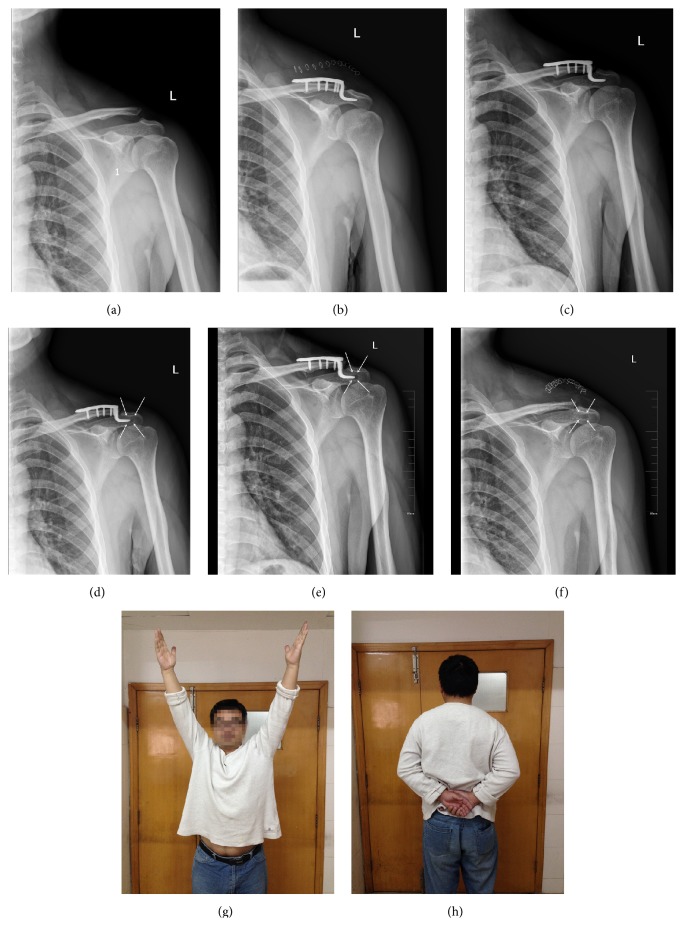
A 30-year-old male patient was diagnosed with left Degree-III acromioclavicular joint dislocation (a) and underwent surgery with a clavicle hook plate (b). The figures show his radiography review two months after surgery (c) and the occurrence of typical subacromial osteolysis nearly five months later (d). The plate was removed at the 10th month after surgery (e-f), and the patient was satisfied with his postoperative shoulder function (g-h).

The mean age of 72 patients was 43.43 ± 12.62 years (range: 16–73 years), and there were 51 male and 21 female patients. 37 patients were injured on the left side and 35 on the right side. The number of patients with a lateral clavicle fracture and AC joint dislocation was 45 and 27, respectively. According to the imaging results, there were 39 patients belonging to group A and 33 patients belonging to group B, respectively. In other words, the incidence of subacromial osteolysis is 54.17% in our study. The age, gender, injury types, and side of the patients in both group A and group B have no statistical difference (Table 1). In the follow-up, we found that patients in group A rarely had subacromial osteolysis within three months. Inversely, this complication usually appeared after 3–6 months and became obvious on the X-ray films after that (Figures 1(a)–1(f)).

Finally, most patients were satisfied with their postoperation shoulder function. More specifically, the mean Constant-Murley scores of the group A and group B were 93.38 (±3.56, 85–100) and 94.24 (±3.60, 86–100), respectively. No statistically significant difference (*P* > 0.05) was found in the scores between the groups as well as the subgroup (Tables 1 and 2).

## 4. Discussion

The shoulder joint is complex in structure and has the largest range of motion of all joints, but easily hurt. Reportedly, the lateral clavicle fracture accounts for approximately 10–15% of all clavicle fractures [2], and AC joint dislocation accounts for 3–5% of all shoulder injuries [1]. Recovering function of the shoulder is necessary following surgery as well as nonsurgical treatment. In a systematic review of 425 fractures, conservative treatment leads to a high nonunion rate (33.3%) [3]. The surgery for patients with Neer II lateral clavicle fracture and Degree-III AC joint dislocation aids in relieving their pain within the shortest possible time, so that patients can do shoulder exercises earlier and avoid complications such as ankylosis and amyotrophy [14, 15]. In recent decades, new techniques have been widely used, such as clavicle hook plate procedure, which has significant advantages in conforming to the anatomical structure and ensuring that intraoperative reduction is easier [4, 8, 13, 16]. Most patients treated with a clavicle hook plate procedure have quite a good prognosis. Apparently, compared to K-wire fixation and Bosworth screw fixation, clavicle hook plate procedure facilitates regaining previous activities earlier [5, 9, 16]. However, there are some inevitable medium-long term complications, particularly subacromial osteolysis, which has a high incidence among the complications and is caused by the design of the clavicle hook [6].

Subacromial osteolysis is not a rare complication of the clavicle hook plate procedure, and its reported incidence significantly differs nationally and worldwide [4–13]. In our opinion, its real incidence may be higher than reported, because, in fact, some patients do not receive long-term and complete follow-up. In our study, the incidence is approximately 54.1% (39 in 72), with no significant difference according to gender or age. This variability in incidence might have several major reasons. Firstly, the time that a plate remains in the body is flexible, as a longer time is usually associated with a higher incidence of subacromial osteolysis. When the plates remained for 12–16 weeks, only 5 patients were identified to have subacromial osteolysis in all 31 samples [17]. In the study of Tambe et al. [10], five of eighteen patients had subacromial osteolysis, who kept their plates in situ. However, there might be other possible reasons, including surgical techniques [12], the patient's race [3, 8, 15, 18], and the hook plate design [6, 8]. Therefore, additional research is needed to determine other potential reasons. Such a high incidence is concerned with its own structure to provide proximal clavicle a sustained pressure; the hook as a pivot of lever under the acromion inevitably bears the stress of several times [6–8]. During the follow-up, we observed that most of the patients in group A suffered obvious osteolysis six to twelve months after surgery, which might be because of postoperative pain and limited activity; meanwhile, due to the relief of pain and increasing exercise, stress has become much more visible, which leads to osteolysis.

The shoulder is the most flexible joint, and the clavicle motion relating to acromion is closely bound up with the raising of shoulder. When the shoulder rises to 90°, the clavicle has a 5° posterior rotation and a 6° upward rotation relating to the acromion; when the shoulder rises to 120°, the clavicle posterior rotation and upward rotation increase to 27° and 21°, respectively [19]. Thus, surgeons should require the patient to avoid activities beyond the postoperative exercise limits. In general, a shoulder that rises less than 90° is acceptable before the plate removal, because under these circumstances, ankylosis and amyotrophy will be avoided and stress under the acromion and abrasion will be decreased [15, 18]. The patients should not return to work too early without medical permission, because their subacromial structures may be damaged [15]; in other words, the patients' compliance is particularly important in rehabilitation.

Additionally, the surgical technique operation is another important cause of subacromial osteolysis. The plate should be selected appropriately and placed to the clavicle as close as possible, and it could be shaped to the clavicle, if needed. Performing anatomical reduction as much as possible in the ORIF (open reduction and internal fixation), surgeons should avoid forcing the clavicle down by the plate in order to decrease the initial stress.

During the follow-up, we find the rate of patients complaint was much lower than the complication incidence, and most patients are satisfied with the postoperative shoulder function (e.g., the male patient in Figures 1(g) and 1(h)). This observation indirectly demonstrates that osteolysis does not cause serious symptoms, which is confirmed by Tambe et al. [10]. Mlasowsky et al. [20] and Hackenbruch et al. [21] similarly used clavicular hook plates in patients and both of them received good results. Flinkkilä et al. used hook plates in 17 patients and compared the results with Kirschner wire fixation and the mean Constant-Murley score in the group following clavicular hook plate fixation was higher than that of the group with Kirschner wires fixation [7]. Moreover, none of these researchers indicated the serious effect of osteolysis.

The plates of the patients in both group A and group B were not removed until ensuring the union of injury. The necessity of removing the plate is the main drawback of this technique because it increases medical cost and necessitates a second operation. An MRI study about the effect of the plate on the subacromial space reveals no increase in rotator cuff lesions; however, it shows an increase in the incidence of extraarticular ossification [22]. Tiren et al. confirms that osteolysis disappears on the follow-up radiographs after the removal of the plates [6]. But once sclerosis is present around the hook, osteolysis will not disappear after the plate removal [23]. The question of whether to remove the hook plate after bony union still remains controversial. Some authors recommend that the plate should not be removed [17, 24]. However, others suggest that the plate should be removed routinely postoperatively to avoid the high possibility of complications occurring [13, 18]. In our hospital, most patients have their plates removed after approximately one year. A limited number of patients retain plates permanently if they do not have any complaints regarding complications and refuse a second operation.

We have to admit that our study only included Constant-Murley after the plates removal and only take subacromial osteolysis into account; other complications were not investigated in this study.

## 5. Conclusion

Subacromial osteolysis is a common complication; however, we preliminarily conclude that in these cases of subacromial osteolysis caused by clavicle hook plate, shoulder function is not significantly affected, regardless of its high incidence, and most patients in our study have achieved satisfied functional prognosis. To sum up, we can regard clavicular hook plate as a considerable treatment for Neer II fracture and Degree-III AC dislocation and try our best to decrease the occurrence of subacromial osteolysis, as well as other complications.

## Figures and Tables

**Table 1:** Clinical data of two groups.

	Group A	Group B	Statistics	*P* value
Age	43.90 ± 12.88	42.88 ± 12.73	*t* = 0.339	0.736
Gender				
Male	27	24	*χ* ^2^ = 0.106	0.745
Female	12	9		
Side				
Right	16	19	*χ* ^2^ = 1.960	0.162
Left	23	14		
Type				
Fracture	24	21	*χ* ^2^ = 0.034	0.855
Dislocation	15	12		

**Table 2:** Subgroup analysis between fracture and dislocation.

	Group A (score/*n*)	Group B (score/*n*)	Statistics	*P* value
Fracture	94.42 ± 4.04/24	93.90 ± 3.19/21	*t* = 0.467	0.643
Dislocation	93.60 ± 3.31/15	94.83 ± 4.30/12	*t* = −0.842	0.408
